# User-centered practicability analysis of two identification strategies in electrode arrays for FES induced hand motion in early stroke rehabilitation

**DOI:** 10.1186/s12984-018-0460-1

**Published:** 2018-12-29

**Authors:** Christina Salchow-Hömmen, Natalie Jankowski, Markus Valtin, Laura Schönijahn, Sebastian Böttcher, Frank Dähne, Thomas Schauer

**Affiliations:** 1 Control Systems Group, Technische Universität Berlin, Einsteinufer 17, Berlin, 10587 Germany; 2 Institut für Rehabilitationswissenschaften, Humboldt Universität zu Berlin, Unter den Linden 6, Berlin, 10099 Germany; 3 Klinik für Neurologie mit Stroke Unit und Frührehabilitation, Unfallkrankenhaus Berlin, Warener Str. 7, Berlin, 12683 Germany

**Keywords:** Functional electrical stimulation, Electrode array, Virtual electrodes, Identification, Hand rehabilitation, User-centered design, Stroke

## Abstract

**Background:**

Surface electrode arrays have become popular in the application of functional electrical stimulation (FES) on the forearm. Arrays consist of multiple, small elements, which can be activated separately or in groups, forming virtual electrodes (VEs). As technology progress yields rising numbers of possible elements, an effective search strategy for suitable VEs in electrode arrays is of increasing importance. Current methods can be time-consuming, lack user integration, and miss an evaluation regarding clinical acceptance and practicability.

**Methods:**

Two array identification procedures with different levels of user integration—a semi-automatic and a fully automatic approach—are evaluated. The semi-automatic method allows health professionals to continuously modify VEs via a touchscreen while the stimulation intensities are automatically controlled to maintain sufficient wrist extension. The automatic approach evaluates stimulation responses of various VEs for different intensities using a cost function and joint-angles recordings. Both procedures are compared in a clinical setup with five sub-acute stroke patients with moderate hand disabilities. The task was to find suitable VEs in two arrays with 59 elements in total to generate hand opening and closing for a grasp-and-release task. Practicability and acceptance by patients and health professionals were investigated using questionnaires and interviews.

**Results:**

Both identification methods yield suitable VEs for hand opening and closing in patients who could tolerate the stimulation. However, the resulting VEs differed for both approaches. The average time for a complete search was 25% faster for the semi-automatic approach (semi-automatic: 7.3min, automatic: 10.5min). User acceptance was high for both methods, while no clear preference could be identified.

**Conclusions:**

The semi-automatic approach should be preferred as the search strategy in arrays on the forearm. The observed faster search duration will further reduce when applying the system repeatedly on a patient as only small position adjustments for VEs are required. However, the setup time will significantly increase for generation of various grasp types and adaptation to different arm postures. We recommend different levels of user integration in FES systems such that the search strategy can be chosen based on the users’ preferences and application scenario.

## Background

Functional electrical stimulation (FES) is a common technique in physical rehabilitation to facilitate the motor recovery of disabled limbs after stroke [1] or spinal cord injury [2]. In therapy, sequences of electrical stimulation pulses are usually applied via surface electrodes to evoke contractions in the paralyzed muscles. The usage of standard hydro-gel surface electrodes has several disadvantages such as lacking selectivity of the stimulation, long placement times, and static electrode positions during therapy sessions [3]. The listed drawbacks are especially relevant for the application of FES on body parts with high muscle density, such as the human forearm, where a selective stimulation is mandatory to generate complex movements (e.g. grasping) [4]. Inaccurate stimulation results and elaborate setup times combined with non-adaptable, open-loop stimulation patterns result in a lack of acceptance and little application of this technology in clinical practice.

Electrode arrays (or multi-pad electrodes) were introduced to overcome these problems and have become popular in FES research within the last two decades [5]. Electrode arrays consist of multiple, small elements, which can be activated separately. By activating multiple elements in a defined temporal pattern (synchronously/asynchronously), so-called virtual electrodes (VEs) can be formed [6, 7]. VEs can dynamically change their position, shape, and size. This facilitates repositioning of the stimulation electrode in real-time by choosing different subsets of active elements.

The application of electrode arrays yields new challenges regarding setup time, VE identification strategies, and user integration. The standard, intuitive manual approach for finding suitable VEs in electrode arrays consists of testing single elements or element combinations iteratively [8]. An element or an element combination is selected, the stimulation intensity is increased until a certain degree of motion is achieved, and the evoked movement is observed and judged by the caregiver. This procedure is repeated until a satisfying VE is found for each desired motion. Nowadays electrode arrays with up to 78 elements are used on the forearm [9]. Thereby, a manual, brute-force search for suitable VEs within electrode arrays is laborious and time-consuming, and additionally may lead to muscle fatigue.

Many approaches have been introduced to automatically find the optimal stimulation point(s) for defined movements within an array. Automatic search algorithms usually combine a stimulation and element testing strategy with a predefined selection criterion, or cost function. In most approaches, the evoked motion via twitch or step stimulation is registered and evaluated in a cost function for each tested element or element combination. Such a function can be the fit with a reference trajectory, which was derived from the movement of healthy people [10], the achievement of predefined constraints for the joint angles [11], or the maximum registered joint angle amplitude [12] together with additional restrictions [13]. The existing methods often evaluate only a small number of stimulation intensities [12, 14] and interpolate the outcome with higher stimulation intensities. Thereby, the search space is reduced, but higher intensities may induce movement in underlying and neighboring muscles as well, which is not considered.

Recently an electro-physiologically based identification approach was suggested which analyzes the FES induced electromyogram of the target muscles to determine suitable VEs [15]. Although the authors presented reliable results, difficulties with this approach are long setup and search times due to the use of multiple electrodes and devices, which will increase even more when complex hand movements shall be generated. Other methods suggest the use of feedback-controlled strategies for the optimization of VEs for hand postures to satisfy the complexity of the problem [16, 17]. However, the current automatic approaches disregard the existing expertise of the users, as the individual opinion of the treating health professional and the patient’s sensing regarding stimulation comfort is often not reflected sufficiently in the decision process. Together with extensive setup procedures, lack of customization, and non-user-evaluated interfaces, this may lead to poor acceptance of electrode-array-based neuroprosthesis in clinical practice. The involvement of users in development processes turned out to increase the usability and acceptance of health care systems such as rehabilitation technologies and is recommended already in early stages [18]. However, systematic usability analyses are missing in current research in the field of array-based FES. One way to overcome these problems is to establish new user array interfaces and dynamic stimulation adaptation via the feedback of integrated sensors, as suggested in [19]. There, individual elements in the electrode array can be activated and deactivated via an overlying touch layer. This allows individual adaptation by the caregiver but can be quite time-consuming.

The scope of this paper is to evaluate array identification methods of different levels of user integration and support to analyze usability, practicality, and acceptance of such methods in clinical early stroke rehabilitation. We present two array identification methods that aim to assist the therapist in finding individual stimulation areas and stimulation parameters according to a patient’s needs and personal training strategy in a convenient way. The first approach is our recently introduced semi-automatic identification procedure which allows the caregiver to continuously modify VEs to find a good stimulation area [20]. The purpose of the semi-automatic search was to provide an identification framework that a) is faster and more convenient than manual search and b) overcomes the lack of user integration and acceptance of fully automatic identification procedures for electrode arrays. In the presented framework, the center of a VE can be modified by the therapist to arbitrary positions within the arrays, and individual stimulation intensities of involved elements are determined automatically with feedback-control.

The second approach is an automatic identification procedure, which identifies stimulation positions as well as parameters, and offers an interactive framework for health professionals and patients. The fully automatic approach might be more appropriate for home use because it provides more assistance in the decision process. This support is especially important for the independent usage by patients. The presented automatic search strategy is examined as an example for the practicability of such approaches in everyday clinical practice. It is an extension and combination of algorithm features from previously published methods by Hoffmann et al. [12] and Schill et al. [14] for hand movements. A cost function is defined on joint angle constraints and calculated for the stimulation of single elements, and element combinations.

Our goal was to validate the two methods for finding suitable VEs and to assess the practicability, effectiveness, and acceptance of the different approaches concerning their varying degree of user integration in a clinical environment. Therefore, we evaluated and compared both methods—the semi-automatic and the automatic approach—in a clinical setup with sub-acute stroke patients for the identification of suitable VEs in a hand neuroprosthesis (HNP). The HNP consists of an electrical stimulator, two array electrodes, two single counter electrodes, and an inertial sensor network for tracking hand motion. The task was to find suitable VEs for hand opening in an array placed above the extensor muscle group in the forearm, and for hand closing in an array covering the hand flexors. Physicians and therapists were instructed to find up to three suitable VEs with each approach for a grasp-and-release motion. After the VE identification, a predefined stimulation pattern was repeatedly applied and the generated hand movement was assessed. Both methods were tested one after another on the same patient with a short break in between, to allow a direct comparison of the results.

For the first time, the application of array search techniques was accompanied by user-centered methods. We conducted face-to-face user acceptance and satisfaction surveys using standardized and system specific questionnaires and interviews. ‘Users’ in the context of rehabilitation systems might refer to patients and health professionals such as physicians and therapists as well [21]. The interests of both user groups need to be considered for the successful integration of new technologies. The following research questions, regarding the functionality and acceptance of the tested array identification strategies, were in the center of our investigations: i) were both array identification approaches appropriate to find suitable VEs?, ii) which approach needed more time?, iii) which approach was favored by clinicians and patients regarding practicability, outcome, comfort, and fun?, iv) what are the essential key-factors for future HNP to gain high impact and acceptance in clinical practice?

Both identification methods are outlined in detail in the following section. Subsequently, the clinical experimental setup and the procedure of the user-centered evaluation are presented. We show results of five sub-acute stroke patients, compare the suggested identification methods, and discuss the relevance of our results for future developments.

## Methods

### Semi-automatic search strategy

**Fig. 1:**
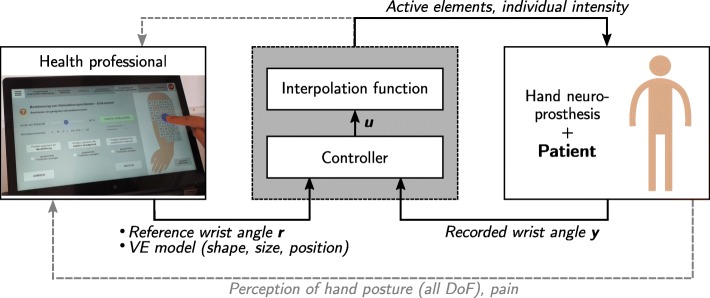
Overview of the closed-loop semi-automatic search. A controller adjusts the global intensity *u* based on the error between the recorded movement of one degree of freedom (DoF) *y* and the reference angle *r* (loop one). The interpolation function assigns an individual intensity to the array elements, which is then applied to the patient. In this picture, a circular VE model is used. Additionally, patient and health professional perceive the other DoF (e.g. individual finger movements) and control the VE model parameters shape, size, and position (loop two)

Common approaches for finding suitable VEs in electrode arrays assess the motion or force that is caused by applying stimulation to discrete positions. Single array elements are either deactivated or stimulated at the same (global) stimulation intensity. Our recently introduced semi-automatic search strategy [20] aims to overcome the restriction of discrete VE positions by providing a smooth interpolation function for the area of the array. The interpolation function determines whether an element should be activated and which individual intensity is applied depending on the position and dimensions of a virtual electrode model in the given array layout. For the model, three different shapes have been realized until now: circle, ellipse, and rectangle. The position of the center of the VE model as well as the dimensions can be changed in real-time by the user, as illustrated in Fig. 1. A graphical user interface (GUI) was developed for devices with touch display. This enables the user to modify the VE model position via finger input (see Fig. 1, *left*) and conveniently test different VE configurations within the array.

Meanwhile, the system supports the user in choosing the applied stimulation intensities via closed-loop control. Our system constantly controls the global stimulation intensity *u*, such that a predefined reference joint angle *r* is achieved in a major degree of freedom *y*. The individual stimulation intensities of the elements rely on the global intensity *u* and can be equal to or less than *u*. The closed loop is depicted in Fig. 1 for the search of a suitable VE for hand extension, as applied in the following experiments. The stimulation is adjusted such that a desired wrist extension is achieved. In this way, the automatic adaption of the stimulation intensity enables the treating health professional to search manually for a sufficient stimulation area, while completely focusing on the current hand posture. The level of applied intensity also serves as information on the current VE parameters. The currently applied intensity is displayed to the caregiver such that he/she can choose the position that yields the desired hand posture with the lowest intensity *u*. However, the closed-loop control is optional and can be deactivated, if desirable. In this case, the global stimulation intensity *u* has to be tuned by the health professionals themselves. For further details on the semi-automatic search strategy including interpolation function and controller design, please refer to [20].

**Fig. 2:**
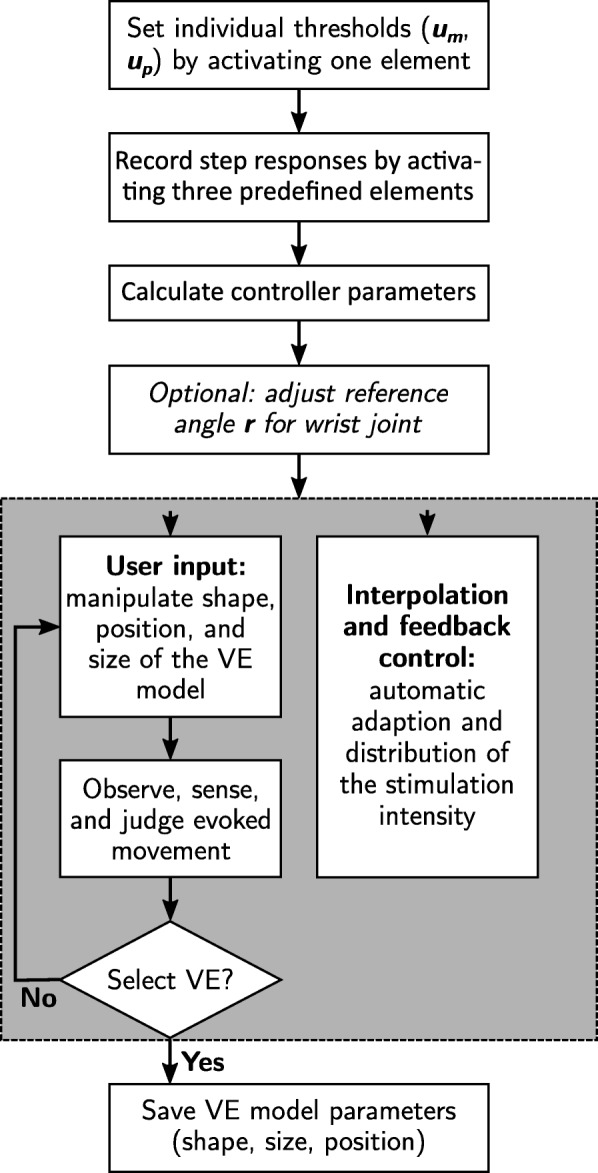
Course of action for the VE identification with the semi-automatic search. After the initialization steps, the user can manipulate the active VE model and observe the resulting motion, while the system automatically adapts and distribute the stimulation intensity (gray box)

The whole procedure of VE identification with the semi-automatic approach as performed in the following experiments is illustrated in Fig. 2. First, motor (*u*_*m*_) and pain (*u*_*p*_) thresholds have to be identified for the individual patient. This was done by stimulating any arbitrary element, representative of the sensation of pain on the forearm [22]. Afterward, step responses of 2 s at three predefined elements, which are distributed across the array, are recorded. The PID controller parameters are calculated based on these measurements according to [20]. The user, in our case the caregiver (physician or physical therapist), can then manipulate the shape, size, and position of the VE model and observe the evoked motion in the patient for DoFs that are not under feedback control. Any position within an array can be tested and saved as a suitable VE for a desired motion. It is possible to combine VEs with different positions to one active VE. In this way, active VEs with branched patterns can be realized, which might be necessary for generating a uniform movement of all fingers [8]. The search with the semi-automatic approach was performed twice, first in the extensor array to find VEs for hand opening and wrist stabilization with feedback control (cp. Fig. 1), and then in the flexor array to find a VE for finger flexion (grasping; see details in the following sections). For the latter, the stimulation intensity applied to the VE in the flexor array was modified manually (open-loop), whereas the already identified VE for wrist stabilization in the extensor array could be stimulated simultaneously in closed-loop mode, to guarantee sufficient wrist extension.

### Automatic search strategy

**Fig. 3:**
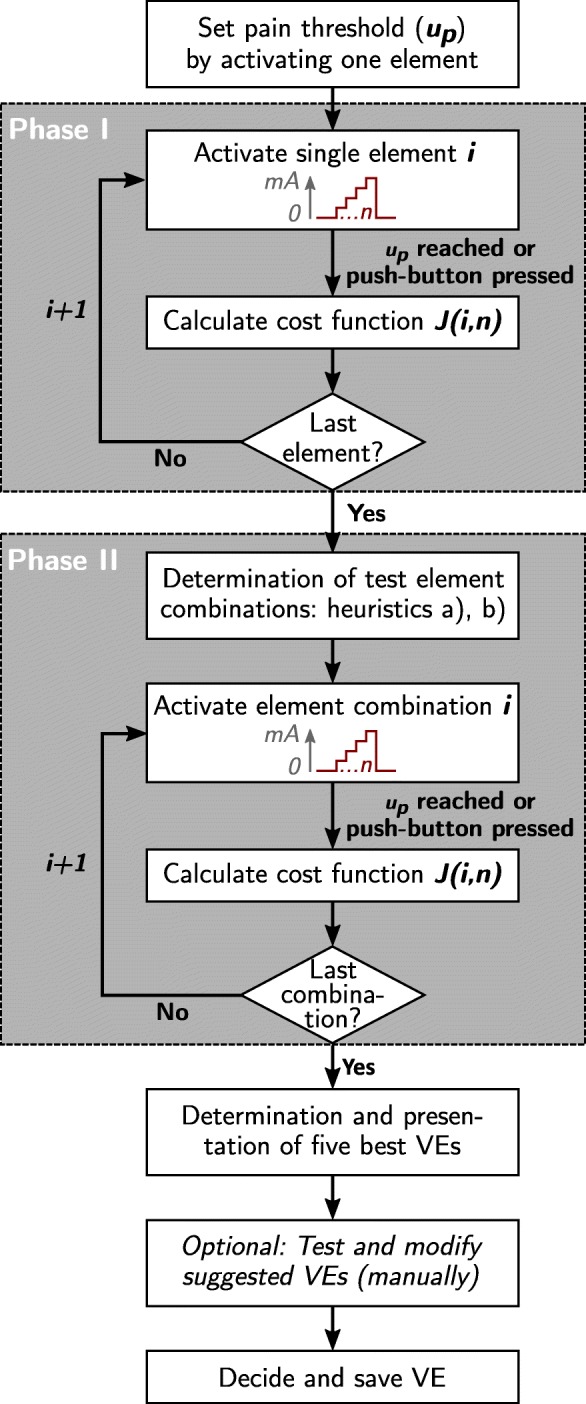
Course of action for the VE identification with the automatic search. First, the maximum tolerated stimulation intensity *u*_*p*_ of the individual patient is determined exemplarily by stimulating one array element manually. Then each element and element combination is stimulated sequentially with a staircase-like profile from zero until *u*_*p*_ (default step-size for the applied normalized charge: 0.01; default step-duration: 120 ms). A push-button was given to the patient, such that he/she was enabled to stop the stimulation at any time. The next element (phase I)/element combination (phase II) is stimulated automatically after 1.5 s of break (adjustable) or the stimulation is started by the patient pushing the button. During phase I, uncomfortable elements can be marked and are excluded for phase II

Parallel to the semi-automatic search, we developed and tested an automatic search strategy as an alternative, which aims to identify suitable VEs and matching stimulation parameters (stimulation current and pulse width). This approach explores the array(s) automatically and suggests suitable VEs for different reference postures. This intelligent procedure might be the right choice when it comes to FES systems in home-use, as it assists the patient in the decision making. The presented automatic search strategy combines algorithm features of previous methods by Hoffmann et al. [12] and Schill et al. [14]. Our algorithm consists of two phases as illustrated in Fig. 3: In phase I, all single elements of an array are sequentially stimulated in a random order with a staircase like intensity profile (single element mode). The induced movement by the electrical stimulation strongly depends on the stimulation parameters (frequency, current, and pulse width). We chose to automatically increase the stimulation intensity (current and pulse width) step-wise for each element until a threshold, such that the induced motion is recorded for varying intensities. The stimulation frequency is set to a fixed value. A cost function *J*(*i*,*n*) based on the observed steady-state joint angle recordings is calculated online after each stimulated element *i* and for each applied intensity level $n \in \mathbb {Z}_{+}^{\ast }$. For each element *i*, the algorithm determines the minimal value

1 $$ L(i) = \min_{n}{J(i,n)}   $$

of the cost function over all applied stimulation intensities. The result of phase I are the four best elements with the lowest *L*. In phase II, element combinations with those elements are formed according to two heuristics and are stimulated in the same manner in an arbitrary order (combined element mode; cf. Fig. 3). Finally, the five VEs with the lowest cost function values over all applied stimulation intensities are suggested as suitable VEs with corresponding stimulation intensities for a given reference movement.

The developed cost function *J*(*i*,*n*) for each element or element combination *i* is defined as follows:

2 $$ J(i,n) = \frac{100}{\sum_{j=1}^{M} g_{j}} \cdot \sum_{j=1}^{M} g_{j} \cdot \|\bar{a}_{j}(i,n) - a_{j,ref}\|.   $$

The function *J*(*i*,*n*) is calculated separately for each of the applied stimulation intensity levels *n* (*n*=1…*N*). With a delay of 60 ms, the recorded joint angles are averaged over the remaining steady-state time interval after each increase of the stimulation intensity yielding $\bar {a}_{j}(i,n)$. The measured and reference joint angles are normalized to the anatomical range of motion of the specific hand and finger joints. The resulting averages $\bar {a}_{j}(i,n)$ are compared with the corresponding reference joint angles *a*_*j*,*ref*_ for each considered joint *j*=1…*M*. The difference of each joint can be weighted individually with the weight *g*_*j*_.

For each desired motion, different reference joint angles and weights can be chosen. It is possible to adapt these values to individual patients. For the experiments conducted in this paper, reference angles have been extracted from recorded hand movements of five healthy volunteers during a grasp-and-release task. The desired movements and matching reference angles can be found in the “Procedure” section.

Two different heuristics were established to build candidate element combinations for phase II. In heuristic a), the element combinations for testing consisted of all eleven possible combinations of the four best single elements (cf. [12]). The maximum number of elements in an element combination selected with heuristic a) thus is four. In heuristic b), the three best single elements are combined with their neighboring elements. Combinations of two elements (good element plus direct neighbor), three elements (good element plus two direct neighbors in a row), and four elements are considered. Combinations with four elements consist of one of the four best elements plus two directly neighboring elements and one direct neighbor of those elements. To limit the number of combinations with heuristic b) and thereby the required time of phase II, an additional selection criterion is applied. Combinations which hold neighbor elements with comparatively high cost function values are excluded. The cost function of an element counted as comparatively high if its value is bigger than the mean cost function value of all tested elements of phase I. The number of all tested combinations of phase II for one cost function is limited to 22, so eleven combinations are selected by each heuristic. For heuristic b), those element combinations are selected which hold the lowest mean cost function over the included elements.

At the end of phase II, the five best VEs of phase I and II are presented to the user (see Fig. 3). Additionally, an array map is displayed showing the distribution of the cost function of the single elements, in other words, the results of phase I. The user is allowed to reexamine the suggested VEs regarding their evoked movement. This manual phase can be necessary because we did not measure all DoF, and to guarantee that the evoked movement matches the expectations of the patient and the treating health professional. However, the users can also decide to trust the system’s decision and simply accept the suggested VEs. Furthermore, new VEs can be built manually by combining elements if necessary. If more than one cost function is investigated, the last three steps of Fig. 3 are repeated for each function/reference posture.

### Experimental setup

**Fig. 4:**
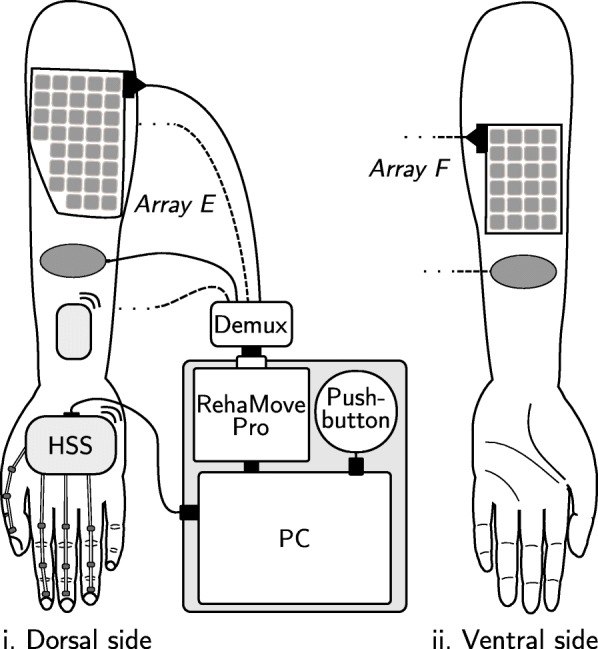
Experimental setup of the hand neuroprosthesis. The setup is exemplarily shown on the left arm

For the clinical validation, we utilized our hand neuroprosthesis consisting of five components, as shown in Fig. 4: The RehaMovePro stimulator with science adapter and demultiplexer (Hasomed GmbH, Germany) [23], two customized electrode arrays with separate counter electrodes, a modular inertial hand sensor system (HSS) for the paralyzed hand [24], a laptop with touchscreen, and an external push-button.

**Fig. 5:**
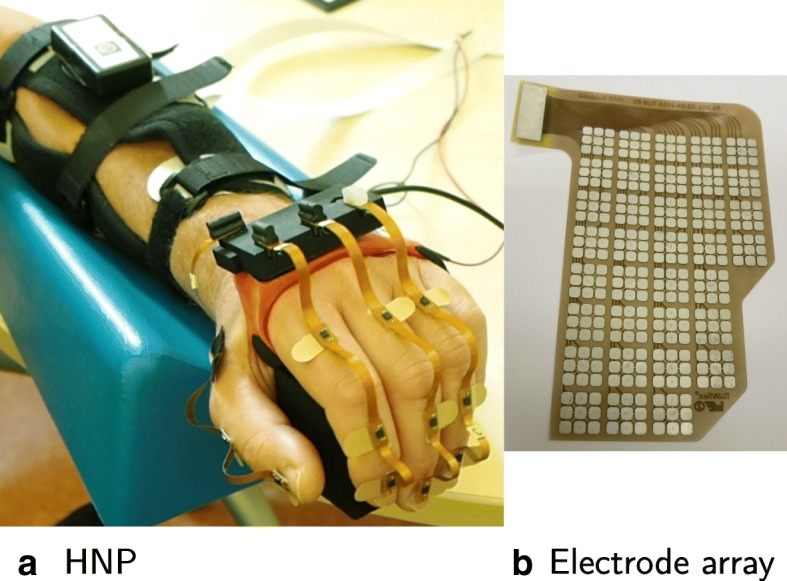
Picture of the utilized hand neuroprosthesis. **a** The HNP is displayed in action on a patient, showing the hand sensor system with four sensor strips on the fingers, base unit, and wireless sensor. The array and counter electrodes are placed beneath the arm cuff. Array E is displayed in detail in **b**

The demultiplexer supports up to 59 active elements and two counter elements. Therefore, one electrode array with 35 elements was designed and placed above the wrist and finger extensor muscles (array E), and one with 24 elements was placed above the finger flexors in the middle of the ventral side of the forearm (array F; cf. Fig. 4). The element size was 12x12 mm^2^ with a spacing of 2 mm. The elements itself consisted of nine connected sub-elements with a size of 3.5x3.5 mm^2^ (see Fig. 5). In this way, we increased the flexibility of the elements and thereby the flexibility and comfort of the whole electrode array. A single hydro-gel layer (AG702 Stimulating Gel, Axelgaard Manufacturing Co., Ltd., USA) was used. The array electrodes were manufactured as flexible printed circuit boards (Würth Elektronik, Germany). In the experiments, the arrays were attached via the gel layers and fixed via a custom-made cuff as seen in Fig. 5. Two oval counter electrodes (4x6.4 cm, ValuTrode, Axelgaard Manufacturing Co., Ltd., USA) were placed at approximately 1 cm distance in distal direction, respectively. Active element configurations could be changed with the frequency of the stimulation, which was necessary for the interpolation in the semi-automatic approach.

Stimulation was applied at 33 Hz as biphasic pulses of asymmetric shape (pulse width, current) but charge balanced. Elements were activated asynchronously, successively after another. Asynchronous stimulation in electrode arrays has been shown to be stable regarding discomfort [22] and to provide benefits on fatigue and selectivity compared to synchronous stimulation [25]. The stimulation pulse intensity *u* equaled the normalized charge of the stimulation pulses. The charge itself is defined as the product of the current amplitude *I* and pulse width *pw* (*u* = 0: *I* = 0 mA, *pw* = 10 *μ*s; *u* = 1: *I* = 80 mA, *pw* = 500 *μ*s). In our setup, *I* and *pw* have been increased or decreased simultaneously while remaining a constant ratio (please see [26] for details).

To track the resulting motion of the paralyzed hand and fingers, we utilized our recently introduced inertial sensor network [24]. The HSS consisted of a base unit with USB connection on the hand back, a wireless inertial measurement unit (IMU) located on the dorsal side of the forearm, and up to five sensor strips for the five fingers. Each strip comprised three 9-D inertial sensors, one IMU placed on each finger segment. We refrained from embedding the HSS in a glove to allow for easy installation on paralyzed hands and to maintain the full sense of touch for the user. Instead, we attached individual sensor strips adhesively to the finger segments with skin-friendly adhesive tape and used a custom-made silicon mount for the base unit. The mounting of the HSS by another person takes approximately 2 min.

In the experiments, we used four sensor strips measuring the thumb (F1), index (F2), middle (F3), and ring finger (F4) (see Figs. 4 and 5). Joint angles were defined in line with the ISB recommendations [27] and estimated via orientation estimation by sensor fusion for each IMU and the calculation of relative quaternions between the connected segments (please see [24] and [28] for details). In total, we measured 19 joint angles with a sampling rate of 100 Hz: extension (negative) and flexion (positive) angle of the wrist (*α*), metacarpal-phalangeal joints (MCP_*α*_), proximal interphalangeal joints (PIP), and distal interphalangeal joints (DIP) of the fingers F2–F4, as well as the abduction angle of wrist (*β*) and MCP joints (MCP_*β*_), and the five joint angles of the thumb (F1). At the beginning of each measurement, the hand with mounted HSS had to remain in a neutral pose for a few seconds during which the heading of all sensor units was aligned. In patients, this pose could be taken up with the help of the health professional.

The (control) algorithms were initially developed in ‘Matlab/Simulink’ (The Mathworks Inc., USA) on a regular PC using a modified Linux ERT target [29]. The GUI was realized in Python and presented on a computer with touch display. During the measurements, the treating health professional was instructed to operate the hand neuroprosthesis via the GUI. An external push-button (PowerMate, Griffin Technology, USA) was given to the patient, such that she/he could interrupt the stimulation at any time.

### Participants

Five sub-acute stroke patients (female=1, male=4, age 53 to 69 (59±6.5), 6–14 days after stroke (8.4±3.2), right-side paralyzed =3) with moderate to moderately severe hemiparesis of the upper extremity were included in the pilot study. Exclusion criteria were severe communication limitations, cognitive dysfunction, and no response towards electrical stimulation at a comfortable stimulation level. The included patients had an mRS (modified rankin-scale) of 3–4 (3.2±0.4) and a muscle strength in the hand and forearm of 0–3 (2.2±1.3) from 5 according to Janda [30]. All patients needed support for performing grasp-and-release tasks with the paralyzed hand.

In addition to the stroke patients as one user group, also the second user group—the health professionals—were included in the user-centered evaluation. The second user group consisted of n=5 (female=1) health professionals (physician=3, ergotherapist=1, medical student=1). Three of five health professionals stated their age with an age range between 33–45 years (39.3±4.9). None of the health professionals had previously used FES in the treatment.

### User-centered evaluation

Both quantitative and qualitative research methods were used to evaluate the functionality and acceptance of the tested array identification strategies as well as our hardware setup from the user’s perspective. Therefore, interviews, questionnaires, and the thinking-aloud technique were utilized to receive a broad insight into their perception of the system and the identification methods. A new method can only be called successful if the technology is accepted by its users, here stroke patients and health professionals (therapists, physicians). A questionnaire for the patients was developed tailored to our system and research questions. The questionnaire contained open-ended and closed questions to gain qualitative and quantitative data. Closed questions were mainly rated on five-point Likert scales from 1 (fully disagree) to 5 (fully agree). The questionnaire for the patients was conducted as an interview and covered sociodemographic data, the personal attitude to technology (measure of technology commitment, [31]), experience with technology in general, usage experience with the system (e.g. problems, understanding, motivation, safety, pain in dealing with the system) and the acceptance of the system via the Technology Acceptance Model (TAM) by Davis [32]. The TAM is one of the most widely used acceptance models. The acceptance and actual use of a technology can be explained in the terms of internal beliefs, attitudes, and intentions of the user, which are decisively influenced by the perceived usefulness and perceived ease of use of the technology.

The concurrent thinking aloud method was used to gain direct information from the health professionals about the interaction with the system. With this method, the participating physicians and therapists are encouraged to verbalize their thoughts continuously while handling tasks with the system (cf. [33]). This should provide access to their thought, feelings, intentions, and expectations and reveal their perception of the actual system use [34].

### Procedure

Measurements were performed at the clinic for neurology with stroke unit and early rehabilitation at the Unfallkrankenhaus Berlin (Germany). If possible, the experiments were conducted with the patient sitting in a chair at a table. Otherwise, the measurements were performed with the patient in a comfortable upright position in their bed. During the identification procedures, the paralyzed forearm was positioned in an arm mount and patients were instructed to obviate voluntary hand movements.

At the beginning of each experiment, the health professionals were familiarized with the thinking-aloud method. In order to gain information about the underlying reasons for their preferred identification method, the health professionals were instructed to express all their thoughts on the system and especially the identification method during the whole session. All sessions were recorded with an audio device. The health professionals had been familiarized with the usage of the HNP in previous workshops and experiments.

Both array identification methods, (A) the semi-automatic and (B) the automatic, were used one after another to find suiting stimulation positions for a grasp-and-release task. A suitable stimulation position was defined in accordance with [35] (“functional point"). The suitable position related to the position/combination of elements of the VE in the array where sufficient strength of contraction can be generated in the target muscles with minimum overflow to non-synergistic muscles. The first method applied was always the semi-automatic search, as the knowledge of the health professional on the stimulation responses gained during the automatic search would have distorted the results regarding search time and positions.

In accordance with [13], three VEs needed to be identified for the grasp-and-release task evoking the following movements: (1) Hand and finger extension for a hand opening (VE_1_), (2) wrist stabilization (wrist extended, fingers in rest or flexed; VE_2_), and (3) functional finger flexion (VE_3_). The corresponding reference joint angles and weights for the cost function of the automatic search strategy are listed in Table 1. VE_1_ and VE_2_ were searched for in array E above the extensors. After a successful identification of these VEs, array F was tested to evoke finger flexion. It was possible to simultaneously stimulate array E for wrist stabilization, even with feedback control for the semi-automatic search. The identified positions, applied stimulation intensities, recorded joint angles, and the duration of each step of the identification procedures were saved for the subsequent analysis.

**Table 1:** Default joint-angle references for the cost function of three VEs

VE	Joint angle references									
	*α*	*g*_*α*_	*β*	*g*_*β*_	MCP_*α*_	*g*_MCP_	PIP	*g*_PIP_	DIP_*ref*_	*g*_DIP_
VE_1_	-20 ^∘^	1	0 ^∘^	0.5	-5 ^∘^	0.25	0 ^∘^	0.25	5 ^∘^	0.25
VE_2_	-15 ^∘^	1	0 ^∘^	0.25	20 ^∘^	0.5	52 ^∘^	0.5	40 ^∘^	0.5
VE_3_	-15 ^∘^	1	0 ^∘^	0.25	20 ^∘^	0.5	52 ^∘^	0.5	40 ^∘^	0.5

The reference joint angles are displayed in degree, the corresponding weights (*g*) have no dimension

**Fig. 6:**
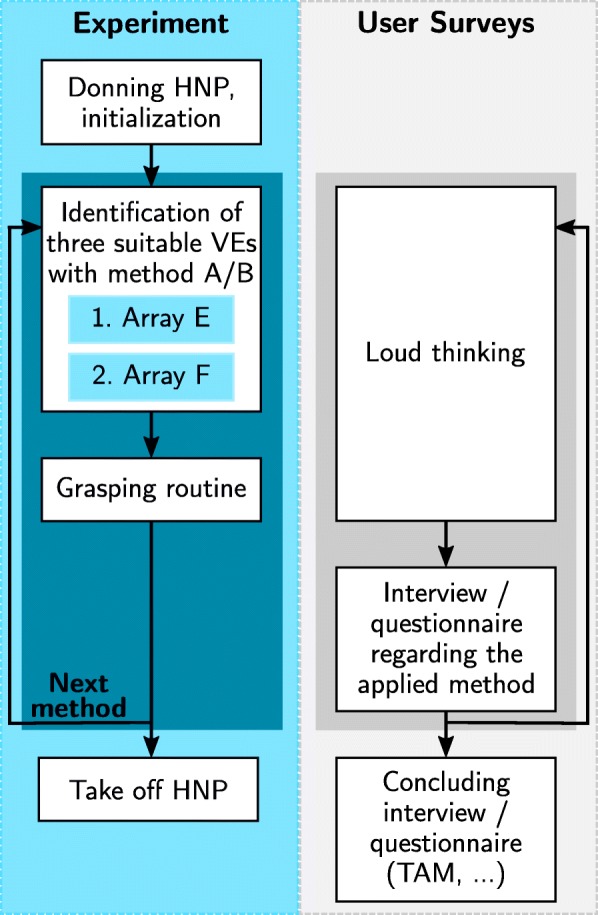
Overview of the experiment and user-centered evaluation. Method A and B refer to the semi-automatic and automatic search strategy

If successful, each identification procedure was followed by a grasping routine, which was performed with and without an object (wooden cube, 5 ×5 cm). The predefined stimulation sequence consisted of 4 s of stimulating a hand opening via VE_1_, then 4 s of stimulating finger flexors (VE_3_) and extensors for stabilizing the wrist (VE_2_; if identified), and ended with 4 s of hand opening (VE_1_). The patient was able to initialize and pause the stimulation sequence via the push-button. It was also possible that the patient controlled the onset and offset of each stimulation phase via the push-button to synchronize it with its own voluntary effort, which was wanted in this state of the experiment. Thereby, different timings within the stimulation sequence (>4 s or <4 s) were possible. If the patient was unable to reach the object due to severe arm palsy, the therapist gave the object to the patient or the object was placed on the table next to the patient’s hand. After each identification approach, the patients were asked about their experiences in a short interview as outlined in Fig. 6, which summarizes the whole experimental procedure.

### Data analysis

*Quantitative*: The subsequent data analysis of recorded joint angles, system parameters, and applied stimulation parameters was conducted with ‘Matlab’ (The Mathworks Inc., USA). Identified VEs were compared between methods and patients. The quantitative data from the interviews of the patients were analyzed with the statistic software ‘SPSS Statistics’ (Ver 22, IBM, USA) using methods of descriptive and nonparametric statistics. To examine if the identification methods cause significant differences in the user’s experience of the system and the stimulation effect, Wilcoxon signed-rank tests were performed. Furthermore, nonparametric correlation analyses according to Spearman were carried out to test, if the user experience is related to age, date and the severity of stroke.

*Qualitative*: To examine the feedback from the health professionals on the identification methods, a qualitative data analysis was performed using strategies of qualitative content analysis by Mayring [36]. The sound material was transcribed according to Dresing and Pehl [37]. In order to answer the research questions, positive and negative feedback to the system and the identification methods, as well as positive and negative feedback to the stimulation outcome was analyzed with the software ‘MAXQDA’ (Ver 12, VERBI GmbH, Germany).

## Results

### Identified VEs

**Fig. 7:**
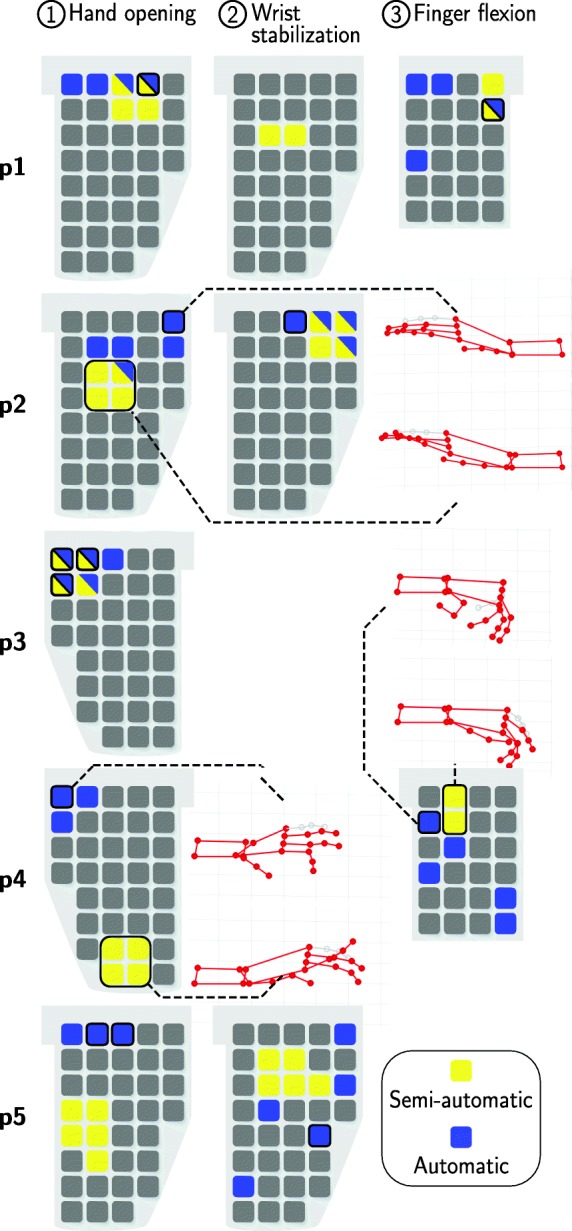
Identified virtual electrodes with the two search methods for each patient. Each row represents the results of one patient for all desired movements, as indicated in the headline. Electrode array layouts are displayed in top view, as positioned on the forearm with gel layer at the bottom. Different array layout orientations are due to the treatment of different arms: right arm for patients 1, 2, and 5; left arm for patients 4 and 5. Search results are marked in yellow for the semi-automatic search and in blue for the automatic search (top five VEs). The finally chosen VE for the automatic search is marked by a black frame. Elements that were identified with both search methods are colored in yellow and blue. For patients and movements where the results of semi-automatic and automatic search are located quite differently in the array the evoked hand motion is depicted (see patient 2 and 3) with the measured hand segments colored in red. If an array layout is not given for defined motion, it means that either no suitable VE was found for that motion (patient 2, 4 and 5) or that the patient could perform the movement on his/her own with the remaining hand function (patient 3)

The identified VEs for all three desired movements in each patient are summarized for both search methods in Fig. 7. Details on the corresponding, identified stimulation parameters are given in Table 2. In all five stroke patients suitable VEs were identified to evoke the movement *hand opening* (VE_1_) with the semi-automatic and the automatic approach. The identified VEs varied in number and position of the active elements for the two methods. VE_1_ consisted on average of 4.2 elements for the semi-automatic search, and on 1.6 elements for the automatic search (cf. Table 2). In general, the VEs identified with the semi-automatic search consisted of more active elements than VEs identified with the automatic search for all three desired movements. For patient **2** and **4**, in which the locations of the chosen VEs were apart for the two methods, the measured hand postures are illustrated in Fig. 7 for stimulating VE_1_ found with the automatic approach (top pictogram) and with the semi-automatic search (bottom pictogram). For both cases, the observed hand posture was similar revealing minor differences in the joint angles of the index finger. It should be noted that the VE for *hand opening* identified with the semi-automatic approach always utilized a VE model of circular shape.

**Table 2:** Details on the identified VEs and corresponding stimulation parameters for both search methods

Patient	*V* *E*_1_							*V* *E*_3_						
	*Semi-automatic*				*Automatic*			*Semi-automatic*				*Automatic*		
	No.	I	pw	Shape	No.	I	pw	No.	I	pw	Shape	No.	I	pw
**1**	4	24.0	244	circ.	1	28.5	289	2	24.9	257	rect.	1	15.5	161
**2**	4	23.0	240	circ.	1	25.0	260	x	x	x	x	x	x	x
**3**	4	18.3	188	circ.	3	22.0	227	x	x	x	x	x	x	x
**4**	4	22.0	222	circ.	1	24.0	250	2	19.1	200	rect.	1	17.0	176
**5**	5	29.5	300	circ.	2	28.5	289	x	x	x	x	x	x	x

The number of involved array elements (*No.*) of the VEs for hand opening (VE_1_) and finger flexion(VE_3_) are listed. *I* denotes the applied stimulation current identified during the identification, here presented in mA. *pw* denotes the corresponding pulse width, here presented in *μ*s. For the semi-automatic method, also the *shape* of the utilized VE model is listed, where *circ.* corresponds to a circular shape and *rect.* marks a rectangular shape. An ’x’ marks cases, where no suitable VE was identified

In two patients (**2** and **5**) suitable VEs were identified to generate a *wrist stabilization* (VE_2_) with the semi-automatic and the automatic approach. In patient **1**, VE_2_ was identified with the semi-automatic search. During its stimulation in the subsequent grasp-and-release pattern, it turned out to hinder a precise finger flexion and was turned off. Hence, a VE for *wrist stabilization* was not considered in the automatic search. Besides the extension of the wrist, the stimulation in the extensor array (E) often led to a small degree of finger extension which hindered the grasping function.

The identification of a position for *finger flexion* (VE_3_) was successful in two patients (**1** and **4**, see Fig. 7). Patient **3** showed sufficient remaining finger flexion such that no FES-support via the flexor array was necessary. The main reason for not finding a suitable VE_3_ in patients **2** and **5** was the low tolerance towards the electrical stimulation in the flexor array. All patients reported the stimulation to be more unpleasant in the flexor array than in the extensor array. This resulted in a lower stimulation intensity maximum, as seen when comparing intensities for VE_1_ and VE_3_ in Table 2, which was sometimes insufficient to evoke a strong finger flexion. Furthermore, parallel induced wrist flexion when stimulating finger flexors was a problem that could not always be compensated by stimulation of VE_2_. In line with the findings for VE_1_, the identified VEs (VE_3_) in patients **1** and **4** varied in number and positions of the active elements for the two identifications methods. For patient **4**, the resulting hand posture is depicted when stimulating VE_3_ found with the automatic approach (top) and with the semi-automatic search (bottom) in Fig. 7. VE_3_ of the semi-automatic search led to less flexion in the wrist. It should be noted that the VE for *finger flexion* identified with the semi-automatic approach always utilized a VE model of rectangular shape (cf. Table 2).

**Fig. 8:**
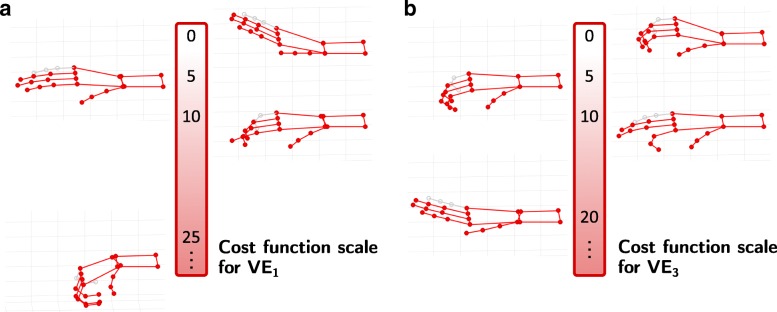
Cost function scale for the references for hand opening (VE_1_) and hand closing (VE_3_). Exemplary hand postures are depicted with corresponding cost function values *J*. For the cost function value “0”, the defined reference postures are depicted for both scales, because a value of “0” means that the generated hand posture equals the reference exactly. The little finger is depicted in gray, because it was not utilized in the experiments and thereby in the cost function. For better illustration, it was assigned the same joint angles as the ring finger

The difference between the identified VEs with both methods was further analyzed by considering the minimal cost function value *L*. For this, the cost function value was calculated offline for the VE of the semi-automatic approach as well. The time frames where the saved VEs were stimulated during the identification process were determined and used for the calculation. The resulting cost function values of both methods are depicted in Table 3. To increase the interpretability of the results, illustrated scales for the references of VE_1_ and VE_3_ are provided in Fig. 8. For VE_1_ and VE_2_ in array E, there is no clear tendency that one method outperforms the other one in terms of the cost function values. The differences were sometimes minor (patient **3**, **5**). In patient **1**, VE_1_ of the semi-automatic search was also stimulated during the automatic search, resulting in a different movement with a larger cost function value (10.4 to 1.8; cf. Table 3), which is why this combination was not chosen in that approach.

**Table 3:** Cost function values for each identified VE with each method for each patient

Patient	*V* *E*_1_			*V* *E*_2_			*V* *E*_3_		
	*Semi*	*Semi in Auto*	*Auto*	*Semi*	*Semi in Auto*	*Auto*	*Semi*	*Semi in Auto*	*Auto*
**1**	1.8	10.4	3.5	11.5	-	x	12.1	-	10.3
**2**	12.0	-	3.1	13.1	8.0	7.9	x	x	x
**3**	1.6	1.6	1.4	x	x	x	x	x	x
**4**	9.9	-	6.0	x	x	x	11.4	-	9.6
**5**	2.3	-	1.6	3.5	-	8.1	x	x	x
**Average**	5.5	-	3.1	9.4	-	-	-	-	-

Here, the abbreviation *semi* refers to VEs identified with the semi-automatic search, and *auto* refers to the automatic search. If the element combination of the semi-automatic VE was tested during the automatic search, the corresponding cost function value is displayed in the column *semi in auto*. If not, this column holds an ’-’. An ’x’ marks cases, where no suitable VE was identified. The average is calculated for columns holding at least three values

### Grasp-and-release task

**Fig. 9:**
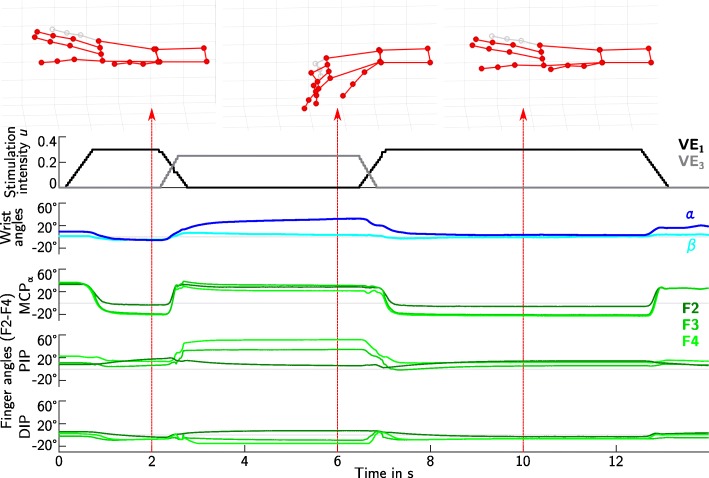
One grasp-and-release cycle with patient 1 using the VEs of the semi-automatic search. The applied stimulation intensities for hand opening (VE_1_) and grasping (VE_3_) are displayed in the first graph. Flexion/extension (*α*) and abduction (*β*) of the wrist are shown in the second graph in blue colors. Flexion/extension angles of the finger joints (MCP_*α*_, PIP, DIP) for fingers F2-F4 are plotted in green in the last three graphs. The measured hand posture including the thumb is visualized at discrete times during the three phases of the grasp-and-release cycle: hand opening, grasp of wooden cube, release. The little finger is depicted in gray, because it was not measured in the experiments. In the interest of a good visualization, the little finger was assigned the same joint angles as the ring finger

Three out of five patients were able to perform the reach-and-grasp task with a wooden cube successfully at the end of the experiment (patient **1**, **2**, and **3**). Figure 9 exemplarily shows the resulting joint angles and hand postures of patient **1** with the VEs from the semi-automatic search. The patient used the push-button to control the timing of the stimulation pattern. This patient was not able to hold the object without the stimulation: finger flexors were stimulated (VE_3_). Patient **2** and **3** were sometimes able to grasp the object without FES-support.

### Search parameters

**Fig. 10:**
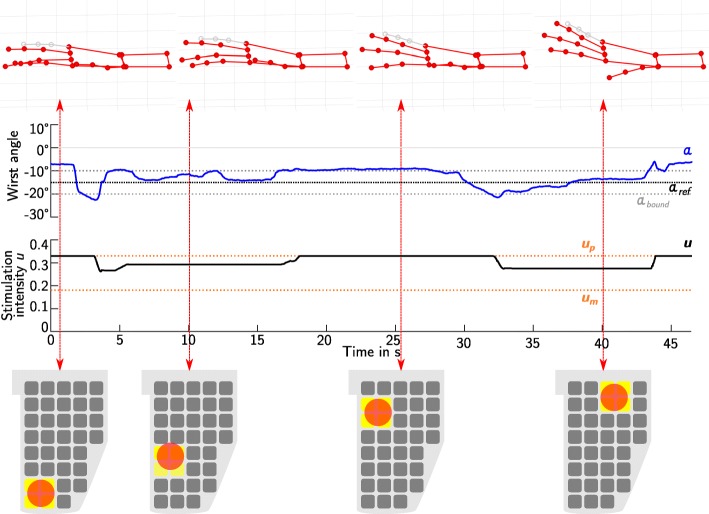
Semi-automatic search in the extensor array with feedback-control for patient 1. The feedback controlled wrist extension/flexion angle *α* (blue) is displayed over time together with the applied global stimulation intensity *u* (black; actuating variable). The reference angle *α*_ref_ was set to 15^∘^ (black dotted line) and the tolerated error bound *α*_bound_ (gray, dotted line) was ±5^∘^. An error smaller ±*α*_bound_ equaled zero at the input of the controller. In the displayed time frame, the location of the VE model was changed by the user. The resulting position of the VE model in the array (red circle) and the corresponding active elements, marked in yellow, are exemplarily shown for four times, together with the measured hand posture. The little finger is depicted in gray, because it was not measured in the experiments. In the interest of a good visualization, the little finger was assigned the same joint angles as the ring finger

In the analysis, we also considered the search process itself. Exemplary results of the feedback control of the wrist angle during the semi-automatic search in the extensor array are depicted in Fig. 10. While the VE model position changed, the global stimulation intensity *u* was adjusted automatically by the controller. As can be seen in this example, there existed positions in the array, where the same degree of wrist extension was achieved with less stimulation intensity. For the depicted patient, a position for *hand opening* (VE_1_) was chosen that needed a lower stimulation intensity than the other positions and led to a strong finger extension. Details on the search settings used in each patient are summarized in Table 4. All provided VE model shapes—circle, ellipse, rectangle—were used during the search process, whereby not every shape was tested in every patient or every array. For the interpretation, it is to be noted that the circular shape was selected by default when starting the identification procedure. The option of combining VEs of different locations to one active VE was not utilized. The feedback control was not applied in patient **4**, because the tolerated level of stimulation was too small to allow closed-loop adaptation. The wrist stabilization via VE_2_ was only used in patient **5** during the search for VE_3_.

**Table 4:** Used options in the semi-automatic search and automatic search for each patient

Patient	Semi-Automatic					Automatic
	*Tested shapes Array E*	*Tested shapes Array F*	*Combined VEs*	*Feedback control (E)*	*Wrist stabil. on*	*Customized VE*
**1**	circular	circular, rectangular	no	yes	no	no
**2**	circular	circular	no	yes/no	no	no
**3**	circular, ellipsoid, rectangular	x	no	yes	x	no
**4**	circular	circular, rectangular	no	no	no	no
**5**	circular	circular	no	yes	yes	yes (VE_2_)

The term *combined VEs* refers to the option of combining VEs with different positions to one active VE. The term *Wrist stabil. on* relates to the option of using the identified VE_2_ for wrist stabilization during the search in the flexor array. In the automatic search, there existed the option on defining *customized VE*, which were not ranked in the top five VE for a desired hand posture. For patient 3, the flexor array was not utilized. The corresponding entries are therefore marked with an ’x’

For the experimental results of the automatic search, we noted that single elements were chosen more frequently than element combinations as the final VE. However, the average cost function value *L* of phase I of the algorithm was always bigger or almost equal to the average value of phase II, indicating that the applied heuristics worked sufficiently. The option of defining customized VEs, which were not rated in the top five by the algorithm for a desired posture, was used once, as seen in Table 4.

### Search duration

**Fig. 11:**
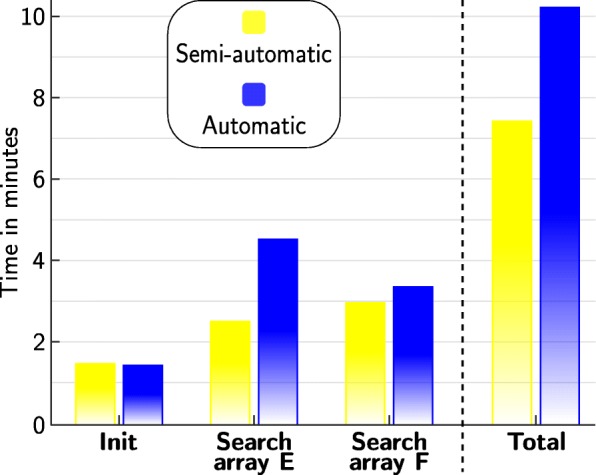
Average duration for the VE search with both identification methods. The time required for the identification procedure is displayed for each period: (i) initialization of the sensors, stimulation thresholds, and for the semi-automatic search the estimation of the controller parameters (Init), (ii) search in the extensor array for VE_1_ and VE_2_, and (iii) search in the flexor array for VE_3_. The sum of all periods leads to the total time needed for each approach (last column)

The donning of the HNP including stimulation electrodes and the inertial sensor network took between 2–4 min. The average time needed for the VE search with each identification method is summarized in Fig. 11. The total duration of the search method included the initialization of the method (adjustment of parameters,...), the search for VE_1_ and VE_2_ in the extensor array, and the search for VE_3_ in the flexor array. In our experiments, the total time of the semi-automatic method (7.5 min) was smaller than the time needed with the automatic method (10.3 min). The search in the extensor array took longer compared to the other periods of the search procedure, which was due to the larger number of elements in that array and the search for two different induced movements (VE_1_ and VE_2_).

### Evaluation by patients

**Fig. 12:**
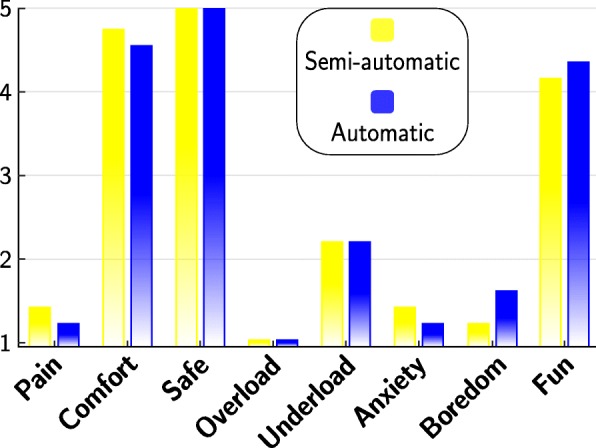
Patients’ perception of the two search strategies. After each identification method, the patients ranked their personal perception in the experiment on a five-stage Likert scale: 1 = strongly disagree, 2 = disagree, 3 = neutral, 4 = agree, and 5 = strongly agree

The survey of the patients after each identification method held similar answers for both methods. Patients were asked regarding the perceived pain on a scale from 0 (= no pain) until 10 (= highest conceivable pain) for the identified VEs. When stimulating in the extensor array (E), average values of 1.2±0.45 were reported by the patients for both methods, stating that only slight discomfort was perceived. For the stimulation of the flexor array (F), the semi-automatic approach led to higher pain values (3.8±3) compared to the automatic approach (2.8±3.5). However, this difference was not significant. In addition, patients were asked after each identification method regarding their personal perception, such as anxiety and fun. They answered the questions on a five-stage Likert scale, as seen in the results in Fig. 12. Again, no significant differences were found between the two methods. When interpreting the results, it has to be kept in mind, that the semi-automatic approach was the first method applied and often was the very first FES treatment of the patients.

The HNP was also assessed in general on a five-stage Likert. On average, the patients agreed that the HNP is comfortable (4.25) and to some extent enjoyable (3.5). The answers regarding technology acceptance were summarized in the dimensions intention of use (4.6), ease of use (4.9), and perceived benefit (5) according to the TAM.

### Evaluation by health professionals

The qualitative analysis of the audio data from the health professionals revealed positive and negative comments regarding the identification methods and the HNP in general. One physician stated the duration of both identification methods as relatively fast but rated the automatic method faster as the semi-automatic method. In the automatic identification method, one physician also indicated to like the random order of the stimulation locations, because of the relieving effect for the muscles. The health professionals made further positive comments, which can be related to both identification methods. In this context, the graphic visualization of the array electrodes and their attachment, as well as the visualization of the current stimulation location in the GUI and the system by itself gained positive feedback. The negative feedback only pertains to the usability of the GUI in both identification methods.

Positive and negative feedback was perceived by the health professionals regarding the outcome of the FES. In the semi-automatic approach, nine statements with positive feedback on the stimulation outcome were determined. The physicians were especially pleased with the stimulated wrist movement. In overall five times, they rated the stimulation effect from “good” to “nearly perfect”. Furthermore, in one test session, the outcome for the index finger was assessed as effective and, in another test session, the physician positively valued the stimulation outcome because of the holistic movement of all fingers. In the automatic approach, 20 positive comments on the stimulation outcome were counted. Four positive statements were related to the overall stimulation outcome and three comments were explicitly about efficient electrode positions. Furthermore, the physicians and therapists remarked in three statements each a good movement effect of 1) the thumb, 2) the index finger and 3) the wrist and gave positive comments on the stimulated hand opening. In two cases, they annotated that the stimulated single electrodes are more efficient than the electrode combinations.

In the semi-automatic approach, four statements including a negative feedback on the stimulation outcome were counted. The physicians indicated the movement of the fingers and especially the thumb as quite weak and stated to see only a small stimulation effect. In the automatic approach, seven statements with negative feedback on the stimulation outcome were found. In two patients, the physicians complained—as well as in the semi-automatic condition—about the missing stimulation effect on the thumb. On the contrary, one participating physician noticed in another patient the opposite effect and criticized that only the thumb and the middle finger showed stimulation effects. Further negative feedback was related to the fact, that the stimulation produced a non-physiological rotation of the wrist.

The participating health professionals gave further clinical indications, which can be used to optimize the system and its handling. Two statements on the importance and the difficulty of the stimulation of the thumb movement were determined. In this context, one physician suggested to move or enlarge the stimulation field (coverage of the array) closer to the direction of the hand.

## Discussion and conclusion

### Summary

We evaluated two array identification procedures with different degrees of user integration, which both aim to assist in finding suitable stimulation areas and stimulation parameters in a hand neuroprosthesis for the individual patient. The results in five sub-acute stroke patients showed that both identification methods–semi-automatic and fully automatic–yield suitable VEs for hand opening and with limitation for hand closing in patients who could tolerate the stimulation. To the best of our knowledge, this was the first time that array identification procedures were directly compared in a clinical setup and the user’s perspective was considered systematically to improve the usability of future FES systems.

### Comparison of VEs

The preferred VEs for hand opening, wrist stabilization, and finger flexion differed among patients, probably due to inter-individual physiological variability and slightly varying array locations on the individual forearm. This finding is in line with other studies (e.g. [10, 13]) and motivates the application of electrode arrays. It also indicates that identification methods need to be applied at least one time for each placed array on the forearm for each patient. Furthermore, in half the cases of our measurements, the preferred VEs with the semi-automatic and automatic approach were found at different areas in the arrays within one patient. We assume several reasons which might contribute to this observation. When considering the generated hand postures with both methods displayed in Fig. 7, and the corresponding cost function values of each VE presented in Table 3, often similar values and postures can be observed. This indicates that there exist several activation areas covered by the array which evoke similar functional movements. Popović-Maneski et al. [35] observed similar phenomena when they identified functional points in hemiplegic patients for a grasp-and-release task. So one reason for the divergent results of the two identification methods is the existence of multiple, equally good solutions in the search space. During the semi-automatic search, the clinician chose a different solution than the automatic algorithm. The term “optimal stimulation point”, as used in other studies on VE identification on the forearm (e.g. [10, 16]), can be misleading.

Another reason for the diverging results of the two methods might be the patient-individual time-variance of the response towards electrical stimulation. This assumption is supported by the cost function values of the VEs identified with the semi-automatic approach. For example, in patient **1**, the identified VE_1_ of the semi-automatic approach was tested within both search procedures and cost function values can be compared. This revealed that the VE_1_ matched the reference nearly perfectly (1.8) during the semi-automatic search, but during the automatic search the same VE yielded a higher cost function value (10.4) indicating a change in the patient’s muscular responsiveness. The duration of the experiment with one identification procedure including explanations, search, grasp-and-release routine, and interview/questionnaire regarding the applied method was approximately 25 min. After this time, when considering the HNP and the patient’s forearm as a system, characteristics might have changed due to reasons such as electrode-skin interface impedance changes, increased muscle tone, or muscle fatigue. This might have led to different selected VEs in the second approach, the automatic search.

Besides the differences in the location of the identified VEs with both methods, the selected VEs also varied in the number of active elements, the shape those elements formed, and in the applied stimulation intensity, as seen in Table 2. We assumed that this was due to the design of the two methods. In the automatic approach, all single elements were tested and thereby also available as VEs to choose. During the semi-automatic search, it was preferred by the users to use a VE model of larger size activating several elements at the same time. In this way, possible activation zones in the array could be manually explored at shorter time. This led to a higher number of elements included in the VEs of the semi-automatic search, 4.2 elements on average, compared to the automatic search with 1.6 elements. It is still an unanswered question whether a lower or higher number of active elements and thereby a smaller or larger VE is beneficial in the therapeutic treatment. Different array layouts in research prevented a direct comparison with findings in other studies. Nevertheless, others identified VEs with a branched pattern [8, 35], which our results do not reflect. Both our methods allow to build a branched active pattern, but the automatic approach requires less effort.

Regarding the stimulation intensities, the values were similar for both identification methods, even though the number of activated elements differed a lot for hand opening (VE_1_; cf. Table 2). A reason for this might be the asynchronous stimulation of VEs with multiple elements. The applied intensities varied between arrays. Stimulation on the ventral side of the forearm was perceived as more painful in general, which was one of the reasons for not finding suitable VEs for grasping in all patients. Sometimes, the tolerated intensity was not sufficient to evoke strong finger flexion as required for manipulating objects. Another reason was the parallel induced but unintended wrist flexion. The stabilization of the wrist by stimulating VE_2_ was often not possible because no stimulation point could be found to exclusively elicit the wrist extensors in the extensor array. Finger extensors were excited as well, which would hinder a successful grasp. Closed-loop control for both VEs (VE_2_ and VE_3_) might be a future solution here, to balance the intensities and thereby the induced motions of both VEs [17, 38]. Yet, closed-loop control requires that patients tolerate sufficient large stimulation intensities.

### Practicability analysis

The results regarding induced motion as well as clinician’s and patient’s feedback indicated no clear preference for one of the two methods. Neither of the identification methods was perceived as painful by the patients, whereby the value of the fully-automatic method was insignificantly better. This might be explained by the fact that the semi-automatic search was always the first method performed and the patients were not used to the sensation of the stimulation at that point. However, the patients felt safe, comfortable, and appropriately challenged during the experiments (cf. Fig. 12). Regarding the HNP, the comparatively poorer rating of the item “enjoyable” to the item “comfortable” with regard to the wearing of the HNP at the end of the experiment could indicate that wearing comfort should be increased for prolonged use of the prosthesis.

The automatic approach received more positive feedback but also more negative feedback than the semi-automatic from the health professionals. The higher amount of comments on the automatic approach could be related to the longer duration of the procedure, with the health professional being less involved. The evaluation of the health professional’s statements on the procedure further revealed that the visualization in the GUI played an important role in acceptance of the methods. Uncertainties were identified regarding the current state of the system, visualization, and operation. A user-friendly GUI, tailored to the individual method and system, turned out to be essential for perception by the users. Therefore, we conclude that future algorithms should always be evaluated in combination with their operation interface for clinical use to deduce their usability and duration in clinical practice, even in early development stages [39].

The average time needed for the VE search was lower for the semi-automatic search than for the automatic strategy. With the donning of the HNP taking between 2–4 min and an average total identification time of 7.5 min for the semi-automatic search, a total setup time for the FES-supported grasp-and-release task of 10 min could be achieved. Most published studies on VE identification did not hold detailed information on the required time of the search procedure in the conducted experiments (e.g. [9, 11, 13, 14]), although it is of high practical relevance. Furthermore, many studies have evaluated only the methodology itself under specific test conditions and not the actual clinical course of action, making it impossible to estimate the needed search time. Bijelić et al. [8] with a manual search (push-button control box) and Popović and Popović [10] with an automatic approach reported a search time of approximately 5 min per 24-element electrode array, which would sum up to >12 min in our setup with 59 elements. Freeman [17] achieved approximately 8 min per posture when using a 40-element array and an iterative learning control approach. Counting hand opening and hand closing as one posture each, this would sum up to 16 min in our setup. According to [10], a duration of <10 min for the phase of electrode-determination is within the level of tolerance for clinical applications. Compared to the mentioned methods, our achieved results with the semi-automatic search for two movements–hand opening and grasping–are faster. However, additional time is needed if different grasp types (e.g. tip grasp) have to be identified and if an automatic adaptation to varying underarm postures (pronation/supination) should be realized.

In rehabilitation therapy, repetitive training with FES is best practice [40, 41]. In previous publications, it was observed that size and shape of individually identified VEs remained the same from day to day in the same patient if the electrode array is placed at the same forearm position (e.g. [10, 13]). In a recent study by Malešević et al. [7], which analyzed the temporal and spatial variability of surface motor activation zones in electrode arrays in 20 FES sessions in stroke patients, it was reported that changes in the VE configuration for wrist, finger, and thumb extension were required each session for all patients. The authors concluded that an experimental (re-)calibration procedure is necessary for each therapy session. They suggested using the results of the previous session as a priori knowledge to reduce the search space in the following session(s). In this application scenario, we conclude that our semi-automatic identification approach would be a suitable tool to gradually modify stored VEs of previous sessions if necessary, which is a benefit in comparison with the suggested and other fully automatic approaches. For the future, we could also imagine a combination of both methods as a suitable approach for clinical practice: In the first session, the whole array is scanned with the automatic approach and the information is saved for the following sessions. Then, the semi-automatic approach is used to individually modify the VEs in the regions of interest.

### Limitations

Changes from day to day in VEs could not be tested in the presented study as the sub-acute stroke patients were only measured once, which is a major limitation of our results. Follow-up experiments would have been desirable, but could not be performed due to the limited time the patients stayed in the neurology department. As most patients used FES for the first time, they were cautious regarding the stimulation intensity and unprepared for the pricking feeling of the stimulation. Nevertheless, the methods were tested in this early stage of the rehabilitation process, because it was suggested to start the treatment of stroke patients as early as possible [42]. Therefore, array identification methods need to prove to be suitable under these conditions.

Another limitation is that the identification methods were tested under restricted conditions, with the forearm lying in pronation on an arm mount. The grasp-and-release task following the identification phase solely included one object without supporting the upper arm via FES. For patients with insufficient, remained voluntary activity in the upper arm, reaching the object had to be supported manually by the caregiver. These limitations have partly been necessary to limit the length of the experiment, allowing a direct comparison of two different methods. As a result, some important features and aspects of electrode array-based FES could not be investigated. Multiple publications mentioned the need for a dynamic VE relocation in the array during forearm movements, which occur in many activities of daily living (e.g. [8, 35, 43]). The rotation of the forearm between pronation and supination yields a relative shift between the active electrode position on the skin and underlying tissue, changing which muscles or motor units are recruited. A real-time adaptation of the active VE in electrode arrays can compensate resulting changes in the generated hand movement, as suggested in [19] or as we presented it in [44]. A clinical validation of this feature is an important aspect of future studies. Especially the setup procedures and identification duration, necessary for the VE identification in different forearm postures, need to be assessed, as it will increase.

That the suitability of the identified VEs was solely assessed in a simplified grasp-and-release task with one object is a major drawback of our assessment. Thereby, the applicability of the method for the identification of different grasp types and strength cannot be estimated. The results in patients **2** and **5** suggest that a re-design of the array electrodes may be needed.

### Conclusions and recommendations

We conclude that both presented array identification methods–semi-automatic and fully automatic–enable for finding suitable VEs in our proposed hand neuroprosthesis. However, the resulting VEs differed for both approaches in three of five patients. The semi-automatic approach should be preferred as the search strategy in arrays on the forearm. The observed faster search duration will further reduce when applying the system repeatedly on a patient as only small position adjustments for VEs are required. Nevertheless, the search duration will increase significantly, when different grasping types shall be generated, or an adaptation to varying forearm conditions shall be realized. It remains to be seen whether this constraint precludes the use in short exercise sessions or whether the repeated use of the system from day-to-day will speed up the identification significantly due to a priori knowledge.

None of the two methods was preferred over the other by the interviewed clinicians and patients regarding practicability, outcome, comfort, and fun. Therefore, we conclude that both levels of user integration should be provided in future FES systems such that the applied method can be chosen individually based on the users’ preferences and the application scenario. Our results underline the need for personalization of the search procedure as, for example, different VE model shapes were utilized during the semi-automatic search and closed-loop support was applied in some patients but not in all.

We found that the design of the GUI influences the acceptance of the methods in general. Further, our results from the patient surveys regarding acceptance and engagement indicate that the motivation of patients at this stage of rehabilitation is particularly high. This observation encourages the application of FES-based neuroprosthesis in early rehabilitation interventions. It follows that the hard- and software must also be evaluated clinically for this type of application. Due to these findings, we recommend the incorporation of end-users in research and product development processes. Future studies in this area should include more detailed questions regarding setup time, handling of the equipment, and desired options in the algorithms for personalization.

For our future system, patient measurements with multiple sessions are necessary to review and maybe re-design the electrode array to generate finger flexion. An additional single surface electrode for the stimulation of thumb (to support grasp) shall be added for future measurements, as health professionals complained about the lack of induced thumb movement in some patients. Furthermore, it is necessary for patients without volitional muscle contractions in the upper arm to support the reaching motion as well. The system presented in the project RETRAINER [45] and the GO-SAIL system [46] are two examples of where an integration of lower and upper arm support was realized. As both presented array identification methods turned out to be suitable for finding VEs for grasp-and-release tasks, both methods together with the forearm movement compensation presented in [44] could be integrated into a holistic system for a hand neuroprosthesis including a user-evaluated interface [21].

## Abbreviations

DIP: Distal interphalangeal joint/angle
DoF(s): Degree(s) of freedom
FES: Functional electrical stimulation
F1: Thumb
F2: Index finger
F3: Middle finger
F4: Ring finger
HNP: Hand neuroprosthesis
HSS: Hand sensor system
IMU: Inertial measurement unit
MCP: Metacarpal-phalangeal joint/angle
PIP: Proximal interphalangeal joint/angle
TAM: Technology acceptance model
VE(s): Virtual electrode(s)
